# Loud Music and Leisure Noise Is a Common Cause of Chronic Hearing Loss, Tinnitus and Hyperacusis

**DOI:** 10.3390/ijerph18084236

**Published:** 2021-04-16

**Authors:** Martin Pienkowski

**Affiliations:** Osborne College of Audiology, Salus University, Elkins Park, PA 19027, USA; mpienkowski@salus.edu

**Keywords:** music, noise, hearing loss, tinnitus, hyperacusis

## Abstract

High sound levels capable of permanently damaging the ear are experienced not only in factories and war zones but in concert halls, nightclubs, sports stadiums, and many other leisure environments. This review summarizes evidence that loud music and other forms of “leisure noise” are common causes of noise-induced hearing loss, tinnitus, and hyperacusis, even if audiometric thresholds initially remain within clinically normal limits. Given the huge global burden of preventable noise-induced hearing loss, noise limits should be adopted in a much broader range of settings, and education to promote hearing conservation should be a higher public health priority.

## 1. Introduction

We operate noisy machines, fire guns, and turn up our music, exposing ourselves to high sound pressure levels (SPLs) with the potential to cause chronic hearing loss, tinnitus (phantom sensations of ringing or other noises in the ears or head), and hyperacusis (discomfort and in some cases long-lasting pain triggered by sound levels that most people can tolerate) [1,2,3,4,5,6,7,8]. The invention of the audiometer and sound level meter [9] enabled research that eventually led most countries, including the United States in the 1970s, to implement legal limits for exposure to workplace noise [10,11,12,13,14,15,16,17]. While these measures (summarized in Section 2) have helped, noise-induced hearing loss (NIHL) and its associated disorders remain the most common injuries in many industrial and military settings, contributing heavily to the huge global burden of hearing loss [18,19,20,21,22,23,24,25,26,27,28,29,30,31,32,33,34]. Regrettably, noise limits have rarely been enforced outside of traditional extraction and manufacturing industries [35]. As detailed in Section 3 of this review, exposure to loud music and other forms of “leisure noise” also commonly causes chronic NIHL, tinnitus, and hyperacusis.

That loud noise can permanently impair hearing and trigger tinnitus has been recognized since antiquity, as mentioned in the writings of Aristotle and Pliny the Elder (tinnitus is Latin for “ringing”). In one of the first issues of The Lancet, Fosbroke [36] wrote that the hearing loss of blacksmiths was “a consequence of their employment, occasioned by the noise of forging; it creeps on them gradually, in general at about forty or fifty years of age. At first, the patient is insensible of weak impressions of sound; the deafness increases with a ringing and noise in the ears…”. Fosbroke was right that noise-induced losses of sensitivity to soft sounds can occur gradually, often becoming apparent only in middle age. However, permanent audiometric threshold shifts (PTS) are also commonly seen in noise-exposed adolescents and younger adults, particularly in the 3–6 kHz “noise notch” range and at the “extended high frequencies” (EHFs), above the 8 kHz limit of conventional audiometry. Fosbroke was also right that tinnitus typically becomes more prevalent and bothersome as “the deafness increases” [37,38,39], but we now know that many people with noise-induced tinnitus, hyperacusis, and difficulties understanding speech, particularly in background noise, have normal or near-normal audiograms [40,41,42,43,44,45,46,47,48,49,50,51,52,53], and may not develop substantial PTS until later in life. Animal lab studies have shown that audiometry is not sensitive to the loss of as many as 30–40% of cochlear outer hair cells [54,55,56,57] and 50–80% of cochlear inner hair cells and nerve fibers [58,59,60,61,62,63]. Thus, considerable damage to the cochlea can accumulate before PTS becomes pronounced, giving rise to the notion of “hidden hearing loss” [64].

Unfortunately, music and noise levels loud enough to permanently damage the ear can for a time feel comfortable and even be addictingly enjoyable [65]. Chung et al. [66] polled a large number of American teenagers and young adults and found that about half had experienced temporary threshold shifts (TTS) and transient tinnitus after attending concerts or nightclubs. If at least some of these events can cause irreparable damage to some fraction of cochlear hair cells and nerve fibers in some individuals—even as audiometric thresholds initially recover, and tinnitus disappears—then enough damage could eventually accumulate with repeated exposures to cause more chronic hearing problems. Indeed, many people attribute the onset of their chronic tinnitus or hyperacusis to a specific loud music or noise dose that did not otherwise stand out from similar experiences in the past. As a young concert goer recently put it: “My tinnitus always went away, until it didn’t. After about a year, I thought, this is permanent now. This is going to suck forever” [67].

## 2. A Brief Summary of Occupational Noise Limits in the U.S.A.

**Figure 1:**
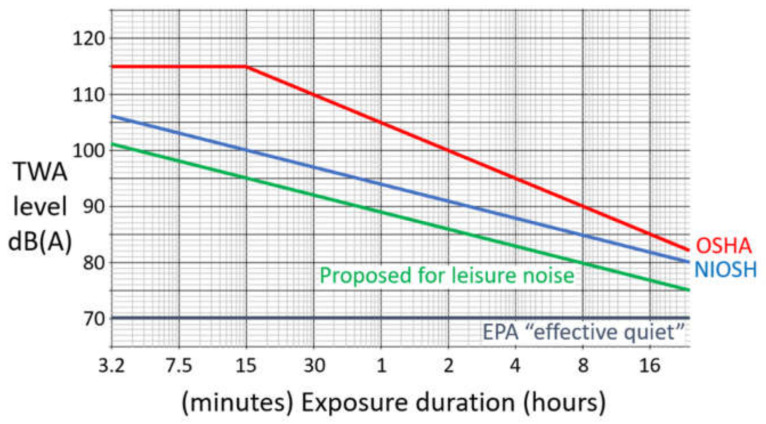
The U.S. National Institute for Occupational Safety and Health maximum time-weighted average (TWA) recommended exposure level (i.e., the NIOSH REL [14] in blue), and the U.S. Occupational Safety and Health Administration maximum TWA permitted exposure level (i.e., the OSHA PEL [16] in red), is plotted as a function of exposure duration. In addition, a recent recommendation for maximum TWA exposure levels for leisure noise [68,69] is plotted (in green), along with the U.S. Environmental Protection Agency (EPA) “effective quiet” level of ≤70 dB(A) [15].

In 1972, the newly formed U.S. National Institute for Occupational Safety and Health (NIOSH) recommended that workplace noise not exceed the time-weighted average (TWA) level of 85 dB(A) over an 8 h workday and 40 h week [14] (The dB(A) scale is a filtered version of the dB SPL scale that compensates for our poorer ability to hear the very low frequencies generated by most noise sources). NIOSH suggested that an additional 3 dB over 85 dB(A) be allowed for each halving of the daily noise dose from 8 h (Figure 1). This 3 dB “exchange” is based on the “equal energy hypothesis”, which maintains that sound energy and exposure duration can be traded off, up to a point, to induce similar TTS. (Note that a doubling of the sound energy represents a 3 dB increase, and so the exposure duration is halved to keep sound energy equal.) For example, the NIOSH recommended exposure limit (REL) for a TWA level of 91 dB(A) is 2 h daily and 10 h weekly.

NIOSH [14] explicitly stated that its guidelines would not safeguard all workers from NIHL. The agency defined “material hearing impairment” as a pure tone average (PTA) threshold exceeding 25 dB HL (hearing level) over the 1–4 kHz frequency range and estimated that a 40-year working lifetime exposure at its REL would cause material impairment in 8% of workers. However, as mentioned above and substantiated below, people can develop tinnitus, hyperacusis, and difficulties understanding speech (especially in background noise) even if their audiogram remains better than 25 dB HL. Moreover, audiometric losses at frequencies above 4 kHz, also not included in the NIOSH definition of material hearing impairment, can by themselves affect sound localization and speech intelligibility [70,71]. Notably, NIOSH [14] cautioned that “a noise capable of causing significant TTS is probably capable of causing significant permanent losses in hearing, given prolonged or recurrent exposures” (Of course, if the noise levels are very high, as in an explosive blast, permanent NIHL can result from a single exposure).

To protect workers against any occupational NIHL—not just the majority of workers from NIOSH’s material impairment—the U.S. Environmental Protection Agency (EPA) [15] derived the much lower 8 h exposure limit of 75 dB(A). However, even this lower limit assumes that workers will not be exposed to additional loud noise from household chores and repairs, hobbies, urban environments, etc. The EPA asserted that sound levels up to 70 dB(A) represent a safe “effective quiet” that poses little risk to people with normal hearing because they do not induce significant TTS [72,73,74]. Unfortunately, noise levels above 70 dB(A) are common in everyday life, on city streets and subways [75,76,77,78], and loud music and noise exposure outside of work undoubtedly exacerbates the risk of occupational NIHL [79,80,81,82,83,84,85].

Despite the NIOSH and EPA recommendations, the U.S. Occupational Safety and Health Administration (OSHA) [16] enforces a substantially higher 8-hour exposure limit of 90 dB(A), with a 5 dB exchange (Figure 1). This means, for example, that the OSHA permitted exposure limit (PEL) for a TWA level of 100 dB(A) is 2 h daily, 8 times longer than the NIOSH REL of 15 min. OSHA caps permitted noise levels at 115 dB(A) for exposure durations of more than one second and at 140 dB pe (peak equivalent) SPL for very brief noise impulses. Although the OSHA PEL is 90 dB(A), employers are legally required to take the following three actions when the 8-h TWA noise level exceeds 85 dB(A): provide hearing protection devices, establish hearing conservation programs for workers that include education and training, and sponsor annual audiometric testing (up to 6 kHz). NIOSH [17] estimated that a lifetime exposure at the OSHA PEL would cause material impairment in 25% of workers, which is close to what epidemiological studies have found [26]. Similar exposure limits and directives have been adopted by the European Union and elsewhere [84,85,86,87,88].

## 3. Loud Music and Leisure Noise Is a Common Cause of Permanent Hearing Loss, Tinnitus, and Hyperacusis

Beach et al. [89] reported that 14% of a large sample of Australian young adults was exposed to an annual leisure noise dose that by itself exceeded the NIOSH REL (18% of 18–24-year-olds, 13% of 25–29-year-olds, and 8% of 30–35-year-olds). This puts the leisure noise problem in perspective. By far, the main culprits, representing ~70% of the total noise energy, were nightclubs (Section 3 ii; see also [81,90]). Similar findings were reported in Michigan’s Kalamazoo County [91]: 15% of adult men and 8% of women were over-exposed by NIOSH criteria to leisure noise alone. Even higher estimates of leisure noise over-exposure have been obtained for teenage and college student populations [81,92,93,94,95,96,97,98,99,100]. The World Health Organization (WHO) [101] recently cautioned that “1.1 billion young people worldwide could be at risk of hearing loss due to unsafe listening practices. Nearly half of all teenagers and young adults (12–35 years old) in middle- and high-income countries are exposed to unsafe levels of sound from the use of personal audio devices, and some 40% of them are exposed to potentially damaging sound levels at clubs, discotheques and bars”. Below we discuss some common sources of loud music and leisure noise and document their effects on hearing. Note that the list is not exhaustive; many of our contraptions can generate sound levels that potentially (depending on the SPL, exposure duration, etc.) pose risks to unprotected ears (e.g., fireworks, power tools, lawn mowers, leaf blowers, hand driers, blenders, etc.).

### 3.1. Live Music Performances and Rehearsals

Audiences at rock or pop concerts are routinely exposed for hours to average sound levels exceeding 100 dB(A) [102,103,104,105,106]. Recall that the NIOSH REL for an exposure level of 100 dB(A) is just 15 min per day, while the OSHA PEL is 2 h per day. For example, Gunderson et al. [105] found that TWA sound levels during rock concerts in eight New York City clubs ranged from 95 to 107 dB(A). A majority of club employees (17/31, 55%) reported acquiring hearing problems on the job, while only 16% regularly wore earplugs. Gunderson et al. [105] concluded that “the development of hearing conservation programs for this large group of workers is essential”. Meyer-Bisch [104] reported that young adults who attended at least one rock or pop concert per month had significantly poorer audiometric thresholds at frequencies ≥3 kHz relative to age-matched controls and were five times more likely to report chronic hearing problems, such as tinnitus. In a sample of people with clinically normal audiograms in the conventional frequency range, more frequent concert-goers exhibited PTS in the EHFs above 8 kHz [107]. Thus, elevated thresholds in the EHFs may be even earlier indicators of NIHL than 3–6 kHz noise notches, a finding corroborated by many other studies reviewed below. Opperman et al. [106] also measured a range of 95 to 107 dB(A) during three rock/pop performances in a popular Minnesotan concert hall. Patrons who did not use earplugs developed an average TTS_2_ (measured immediately after exposure) of 10 dB from 3 to 6 kHz, but this varied widely (SD = 10 dB; see also [108]), whereas the small minority of patrons who did wear earplugs did not develop significant TTS. Bogoch et al. [109] reported that 85% of a rock concert audience experienced transient tinnitus after the show, while only 20% wore earplugs.

Axelsson and Lindgren [110] found that almost half (46%, 38/83) of surveyed Swedish professional rock/pop musicians exhibited some PTS in the conventional frequency range (threshold ≥20 dB HL at least at one frequency). In a follow-up study of the same musicians 16 years later, Axelsson et al. [111] reported that their hearing loss had become worse compared to age-matched controls, especially at 4 and 8 kHz. Similar results on a different sample of 139 Swedish professional rock/pop/jazz musicians were published by Kähäri et al. [112]: 49% had a PTS in the conventional frequency range (using the more stringent criterion of ≥25 dB HL at two frequencies, or ≥30 dB HL at one frequency), and 74% reported tinnitus, hyperacusis, and/or diplacusis (the perception that a single sound has a different pitch in the two ears). Juman et al. [113] found that noise-induced audiometric losses in steelpan drummers tended to emerge slowly, becoming significant relative to age-matched controls only after age 40. Nevertheless, two-thirds of drummers who played for >20 years developed significant PTS, as did 40% of drummers who played for 10–20 years [113]. Schmuziger et al. [114] found that a sample of 42 amateur rock/pop musicians, drummers, in particular, had poorer than expected thresholds at 3–8 kHz; 26% were diagnosed with hyperacusis and 17% with tinnitus. Størmer et al. [115] also noted that Norwegian rock musicians had a PTS at 3–8 kHz compared to controls, and 20% had chronic tinnitus. McIlvaine et al. [116] had members of a rock band wear personal dosimeters during a typical 2 h rehearsal and 4 h performance. The rehearsal exceeded the NIOSH REL but fell within the OSHA PEL, while the performance exceeded even the OSHA PEL, underscoring the risks of performances in particular to rock musicians’ hearing.

Average sound levels in a symphony orchestra pit also typically exceed 85 dB(A) during rehearsals and performances [117,118,119,120,121]. While orchestral musicians do not play together for 40 h per week year-round, about 50% exceed the weekly NIOSH REL during solitary practice alone [122]. Nevertheless, one early study of Swedish orchestra musicians by Karlsson et al. [123] reported no evidence of NIHL and concluded that “sound exposure criteria for industrial noise are not valid when discussing such sounds as are produced by acoustic instruments in a symphonic environment”. In support of this conclusion, Strasser et al. [124] found that exposure to 2 h of classical music at an average level of 91 dB(A) (the NIOSH REL) induced much less TTS (~10 dB) than the equivalent dose of industrial noise (~25 dB) in the same subjects. Strasser et al. [125] had earlier shown that there was no such difference between industrial noise and heavy metal music. Classical music is typically more spectrotemporally modulated than heavy metal music and industrial noise, and perhaps the emotional response to classical music also has some otoprotective effect [126].

Even if loud classical music poses a lower risk of NIHL than a similar dose of heavy metal music or industrial noise, the negative result of Karlsson et al. [123] has been overturned by many subsequent studies [118,120,127,128,129,130,131,132,133,134,135,136,137,138,139,140]. For example, Royster et al. [118] found that Chicago Symphony Orchestra musicians were exposed at just above the NIOSH REL during their 15 h of weekly rehearsals and performances (not counting solitary practice). About half of these Chicago musicians (53%, 31/59) had audiometric notches at 3–6 kHz, and violinists and violists had poorer 3–6 kHz thresholds in their left compared to right ears, consistent with the left ear receiving greater exposure from their instruments [118]. Noise notches were also found in musicians employed at the Gothenburg Symphony Orchestra and the Gothenburg Opera in Sweden [130,131], and in 45% (149/329) of American college student classical musicians [134]. Student musicians with normal audiograms (no noise notches) in the conventional frequency range nevertheless had poorer EHF thresholds compared to controls [141,142] (but see [143] for a negative finding), reported more loudness tolerance problems (i.e., hyperacusis), and exhibited poorer speech intelligibility in noise [142]. A study of average sound levels associated with high school and university marching bands, which are especially popular in the U.S.A., reported a range of 95–122 dB(A) for drum sections and 106–118 dB(A) for cymbal sections, both greatly exceeding the NIOSH REL [144] (see also [145,146]). However, the only audiometric study on university marching band members that I could find failed to detect evidence of PTS in the conventional frequency range [147].

Clark [103] measured average sound levels below 90 dB(A) in the audience of classical music concerts, remarking: “Given that even the most enthusiastic concert-goer most likely could not exceed 20 h per week of exposure, it is unlikely that attending classical music concerts poses any risk of NIHL for anyone”. However, Clark [103] noted that audiences of big jazz ensembles could be exposed to potentially more threatening levels, averaging around 95 dB(A).

In a meta-analysis of 41 studies counting 4618 professional musicians, Di Stadio et al. [148] reported that 64% of rock/pop musicians had at least some audiometric loss, compared to 33% of classical musicians. The prevalence of chronic tinnitus was 26% in both rock/pop and classical musicians, while that of hyperacusis was 27% in rock/pop musicians and 19% in classical musicians [148]. Many high-profile rock/pop musicians have come forward with sad personal stories and warnings about NIHL, tinnitus, and hyperacusis [149]. In a recent landmark legal case, London’s Royal Opera House was successfully sued by a violist who was exposed to peak levels of 132 dB(A) during a rehearsal of Wagner’s Die Walkure and afterward began to suffer from debilitating tinnitus and hyperacusis [150]. The Opera House appealed the verdict, claiming that the artistic value of the music meant that some hearing damage to its performers was inevitable and justifiable, but this was rejected by the Court of Appeal, which maintained that the orchestra pit should be subject to the same noise regulations as a factory floor.

### 3.2. Discotheques and Nightclubs

Tan et al. [151] recorded sound levels in 20 Hong Kong discos and reported a grand average of 95 dB(A) that was quite consistent across venues. Employees of these discos typically worked more than 50 h per week and so were exposed at substantially above the NIOSH REL [151]. Similarly, high sound levels were subsequently recorded in nightclubs in Singapore [152], the U.K. [153], South Korea [154], the U.S.A. [155], Germany [156], and Ireland [157].

Potier et al. [158] studied the hearing of 29 young professional disc jockeys (DJs) who worked in French clubs for an average of 6 years. In addition to the usual 3–6 kHz noise notch, they tended to have hearing loss at frequencies below 1 kHz (likely from cranking the bass), and 76% reported chronic tinnitus. In an important 4-year longitudinal study, Argentinian teenagers who occasionally attended discos developed PTS at 14 and 16 kHz to a greater extent than at 4 and 6 kHz [93,159,160,161], again suggesting that hearing sensitivity at the EHFs is most vulnerable to noise trauma. Johnson et al. [98] surveyed 325 British university students and reported that 88% experienced tinnitus after leaving a nightclub, and 66% still noticed TTS the following morning. Although over 70% felt that nightclub noise should be limited to safe levels, a similar percentage claimed that they would continue attending clubs despite knowing the risks of NIHL.

### 3.3. Personal Listening Devices

Billions of people of all ages listen to music via earphones paired to cell phones or other personal listening devices (PLDs). Some listen at dangerously high levels that can average well over 100 dB(A) at maximum volume settings [162,163,164], which puts users at risk of NIHL [165].

Fligor et al. [166] found that 60 adult residents of boisterous New York City set their PLDs at the average level of 94 dB(A); 62% of them exceeded the daily NIOSH REL with PLD use alone. Similar numbers were reported in a larger sample of 189 New York City college students: 58% and 52%, respectively, exceeded daily and weekly NIOSH limits through PLD use alone [167]. Such high music levels are typical when trying to drown out loud background noise [163]. In ostensibly quieter Australian environments, Gilliver et al. [168] reported that about 10% of PLD users exceeded the NIOSH REL. For Israeli teens, that number was about 25% [169], while 44% of Canadian teens self-reported loud PLD use [81]. Primary school children also appear vulnerable to NIHL from PLD use, especially boys [81,170,171] (see also [172,173] for other loud toys).

Peng et al. [174] found that Chinese university students who used PLDs had poorer audiometric thresholds above 3 kHz compared to controls, with the greatest differences above 6 kHz. Additionally, PLD users with clinically normal audiograms to 8 kHz had elevated EHF thresholds [174]. Le Prell et al. [175] also found elevated EHF thresholds in young American adults with clinically normal audiograms who reported long-term or high-volume PLD use. Elevated EHF thresholds despite normal conventional thresholds, as well as reduced transient evoked and distortion product otoacoustic emissions (TEOAEs and DPOAEs), were also found in a group of PLD users with otherwise unremarkable noise exposure histories by Sulaiman et al. [176].

### 3.4. Non-Motorized Sports

According to the Guinness Book of World Records, the loudest crowd roar recorded in a sports stadium reached 142 dB(A) at an NFL (National Football League) game at Arrowhead Stadium in Kansas City, Missouri, on 29 September 2014. Engard et al. [177] had 28 workers and 25 fans wear personal dosimeters in medium and large college American football stadiums and NFL stadiums, with average game attendances of ~20 K, 50 K, and 75 K, respectively. TWA noise levels were similar across games and stadiums despite the varied attendances, ranging from 91 to 95 dB(A); almost all sampled workers and fans were overexposed by daily NIOSH criteria [177]. Engard et al. [177] concluded: “Facility managers should include a warning in fan guides, pamphlets, websites, or other appropriate communication tools of possible loud-noise exposure during any sporting events held at the stadiums. This information should include the health effects of loud noise exposure, namely, noise-induced hearing loss. The information also should be specifically targeted to parents of young children, including a strong recommendation that hearing protection should be worn by all children during the sporting event”.

Hodgetts and Liu [178] recorded TWA levels of 101–104 dB(A) in Canadian hockey arenas during NHL (National Hockey League) playoff games, with peaks above 120 dB(A). During collegiate and minor league professional hockey games in American arenas, average sound levels were almost 90 dB(A) [179]. Swanepoel and Hall [180] reported that noise levels at a South African Premier Soccer League match averaged 100 dB(A) and that spectators incurred TTS and DPOAE amplitude reductions measured after the match. The games of the 2010 FIFA (Federation Internationale de Football Association) World Cup, also held in South Africa, were even louder due to the prolific use of vuvuzelas, African horns which generate sound levels of about 130 dB(A) at the horn opening. Flamme and Williams [181] found that Michigan sports officials had a greater prevalence of self-reported hearing difficulties, including tinnitus, than the general population; sound levels produced by whistles, which ranged from 104 to 116 dB(A), were noted as potential contributing factors.

Torre and Howell [182] recorded average music levels of 87 dB(A) during 50 min aerobics classes and found that these caused small but significant transient reductions in DPOAE amplitudes. Nassar [183] found that all participants in an aerobics class developed significant TTS after a 1 h exposure to 92 dB(A). Beach and Nie [184] measured TWA levels of 93 dB(A) in high-intensity aerobics classes and reported that only 20% of participants said that such loud music was stressful and that 85% of instructors considered it to be motivating.

### 3.5. Motorized Sports and Hobbies

Rose et al. [185] measured noise levels during a NASCAR (National Association for Stock Car Auto Racing) race and reported that they ranged from 99 to 109 dB(A) 20 feet from the track and 96–104 dB(A) 150 feet from the track. In the pit area, peak levels exceeded the OSHA limit of 140 dB(A) [185]. Kardous and Morata [186] found that race car drivers and pit crew members were exposed to noise well above the daily OSHA limit. Hearing protection use was variable and intermittent among team members [186]. Morley et al. [187] measured average sound levels of 95–100 dB(A) at monster truck and motocross shows. McCombe and Binnington [188] surveyed 44 young motorcycle Grand Prix racers and found 45% had some audiometric loss, while only nine riders (20%) were regular earplug users.

Ross [189] measured noise levels inside five models of full-face motorcycle helmets at a riding speed of 60 mph and reported a range of 95–103 dB(A), with a spectral peak between 250 and 500 Hz (see also [190]). McCombe et al. [191] obtained audiograms from 246 motorcyclists with otherwise unremarkable noise exposure histories and found that they had hearing loss predominantly at 500 and 1000 Hz, as expected from the helmet noise spectrum. Moore [192] measured in-helmet noise levels for 10 recreational snowmobilers during rides averaging a distance of about 50 miles and found that all riders exceeded the daily NIOSH REL.

### 3.6. Rifle Shooting

Gunfire from pistols, rifles, and shotguns produces peak sound levels of ~135–175 dB(A) [102], typically exceeding the NIOSH and OSHA limit of 140 dB(A) for impulse noise, which is an especially dangerous noise type [193]. In a classic study, Taylor and Williams [194] compared the audiograms of 103 middle-aged sports hunters to age-matched controls and found that the hunters had 30–40 dB worse thresholds at 3–6 kHz in the left ear. In the right ear, which had been partially acoustically shielded by shouldering the gun on the right side, the thresholds were only 10–20 dB worse. If the partial shielding of the right ear with the stock of a gun was enough to prevent much of the apparent NIHL, it almost certainly could have been prevented entirely had the hunters worn suitable hearing protection (Section 4). In a more recent study, Stewart et al. [195] reported a similarly high incidence of hearing loss in waterfowl hunters and a high prevalence of tinnitus; 88% of hunters were aware that firearm use could cause hearing loss and tinnitus, but many still chose not to use hearing protection while shooting.

Stewart et al. [196] studied the hearing of 210 young (10–17-year-olds) recreational firearm users and found that while most had clinically normal conventional audiograms, 10% reported chronic tinnitus and 45% transient tinnitus after shooting. Most kids (81%) were aware that shooting could result in hearing loss and that hearing protection should be used, but only 56% reported using it consistently during practice shooting, and only 16% while hunting.

## 4. Preventing Noise-Induced Hearing Loss

As mentioned in Section 2, lifetime noise exposure at the NIOSH REL (85 dB(A); 3 dB exchange; 40 h/week) and OSHA PEL (90 dB(A); 5 dB exchange; 40 h/week) is estimated to cause “material hearing impairment” (1–4 kHz PTA ≥ 25 dB HL) in 8% and 25% of workers, respectively [17,34]. Many more people whose conventional audiograms remain better than 25 dB HL develop other symptoms of NIHL, including difficulties understanding speech, especially in background noise, and chronic tinnitus and hyperacusis [40,41,42,43,44,45,46,47,48,49,50,51,52,53,197]. As shown in Section 3, loud music and leisure noise doses often exceed NIOSH and even OSHA limits, putting workers of concert venues, nightclubs, and sports stadiums, as well as the attending public, at considerable risk of NIHL.

While conventional audiometry fails to capture the true extent of NIHL and its associated problems, it appears that even audiometrically-measured hearing loss in young people is on the rise. Shargorodsky et al. [198] analyzed the large-scale U.S. National Health and Nutrition Examination Survey and found that the prevalence of all-cause hearing loss in teenagers increased from 14.9% in 1994 to 19.5% in 2006. Hearing loss was categorized by either a low-frequency (0.5, 1, 2 kHz) or, more commonly, by a high-frequency (3, 4, 6, 8 kHz) PTA that exceeded 15 dB HL in one or both ears [198]. Using the same criteria, the prevalence of hearing loss in Korean teens in 2016 was about 17% [199]. Although some have questioned the use of this 15 dB HL PTA cutoff [200], Shargorodsky et al. [198] reported that the prevalence of more severe hearing loss (PTA > 25 dB HL) in U.S. teens also increased, from 3.5% in 1994 to 5.3% in 2006. It seems likely that repeated exposure to loud music and leisure noise is a major reason for this increase.

A complicating factor in establishing safe noise exposure limits is the large difference in susceptibility to NIHL between individuals [201,202]. Males are more susceptible than females, not just because they are generally exposed to more noise but because the hormone estrogen (expressed in higher levels in females) is otoprotective [203]. Melanin is also otoprotective, so people with fair skin and eyes are more susceptible to NIHL than those with dark skin and eyes [204,205,206], as are people with diabetes [207], Bell’s palsy (who lack an acoustic reflex) [208], and those with more efficient outer and middle ears [209,210]. Common genetic mutations associated with cochlear antioxidant defense systems can also increase vulnerability to NIHL [211,212,213]. For example, Chinese factory workers were at higher risk for NIHL when they carried a point mutation in the mitochondrial Mn-superoxide dismutase gene [212], compromising their ability to effectively neutralize mitochondria-generated superoxide, which is toxic to cochlear hair cells if not scavenged rapidly [211]. Finally, although the risk for permanent damage to cochlear hair cells and nerve fibers increases with the amount of TTS induced by a given noise dose, TTS is not a strong predictor of PTS at specific frequencies in individual ears [201,202,214,215].

Recent analyses have recommended that exposures to leisure noise not exceed an 8-h TWA level of 80 dB(A), with a 3 dB exchange [68,69] (Figure 1). Under these guidelines, a two-hour concert should be enjoyed at a level no greater than 86 dB(A), assuming that is the only dose of loud sound for the day. Numerous smartphone apps, some free and most others available for just a few dollars, can be used to accurately track sound levels, especially when they are calibrated with a sound level meter. This author has recorded sustained sound levels in the potentially hazardous 85–105 dB(A) range at dozens of school dances and talent shows, amateur sports competitions, outdoor car and bike shows, bars and clubs, and in many other common recreational settings. Quite simply, we should build and use quieter machines, turn down the volume to reasonable levels (80 dB(A) or lower), and shun louder exposures whenever possible. Music and noise loud enough to induce tinnitus, no matter how temporary, should be avoided, although further research is needed to confirm that transient tinnitus is a reliable indicator of permanent (if for a time hidden) hearing loss. When avoidance is not possible, as in the symphony orchestra pit, for example, the use of hearing protection should be strongly advised if not mandated.

Even cheap foam earplugs are often sufficient to attenuate excessively loud music and noise down to safer levels, although foam plugs distort sound by providing much more attenuation at high compared to low frequencies. For as little as $5–10 USD per pair, “musician’s earplugs” provide much more uniform attenuation across frequency, allowing for nearly distortion-free listening, and for some people, even improved speech understanding in loud background noise [216,217,218]. Another option is an active noise-canceling headphone [219,220,221]. Stage musicians can protect their ears while improving their ability to hear their music by wearing “in-ear monitors”, which are Bluetoothed to the amplification system [222]. Double hearing protection, typically consisting of foam earplugs worn under over-the-ear muffs, should be used in the loudest environments, when average 8-h exposures exceed 100 dB(A), and when impulse noise exceeds 140 dB pe SPL, as when shooting guns [17,223].

Despite the many good options for protecting the ear from excessively loud music and noise, usage in non-work environments remains low, especially among young people [224,225,226,227,228,229,230]. Some of the stigma associated with earplug use can be attributed to ignorance or lack of concern about NIHL. Raising awareness at an early age should be a priority, yet despite decades of efforts [231,232,233,234,235,236,237,238,239,240,241,242,243,244,245,246,247] (Table 1), basic information to promote hearing conservation remains absent from most school curricula. Hearing conservation should be given similar attention and resources to those allocated for other school health education programs, such as anti-smoking/drug/alcohol programs or sexually transmitted disease and teen pregnancy programs. Some school boards screen children for hearing impairments and such screening could be expanded to include EHF audiometry and DPOAEs, establishing more sensitive baselines for the early detection of future NIHL.

NIHL is currently a permanent disability. A number of pharmacologic interventions to help protect the ear from over-exposure to loud noise appear to be on the horizon, but nothing is FDA-approved yet [248,249]. Hearing aids are helpful but do not restore natural hearing ability, and there is currently no cure for tinnitus or hyperacusis [250], nor for bringing back lost cochlear hair cells and nerve fibers [251,252,253]. NIHL can precipitate depression, social withdrawal, and cognitive decline [254,255,256,257,258,259,260], and mild, moderate, and severe hearing loss increases the risk of dementia by 2-, 3- and 5-fold, respectively [261,262,263]. For many people with hyperacusis, ambient noise levels in restaurants, malls, and on city streets can be intolerably loud. Severe tinnitus and hyperacusis can drive people to suicide [264].

The seemingly indomitable Yul Brynner, like so many others before and since, publicly lamented his chain-smoking habit prior to his untimely death of lung cancer. Like smoking and lung cancer, NIHL is highly prevalent, has far-reaching consequences, and is largely preventable. The relatively recent smoking bans in many indoor and outdoor public spaces represent major public health victories that the hearing field should emulate. While more basic and epidemiological research into safe vs. unsafe noise levels is needed (a notable gap in the literature described in Section 3 is the hearing status of athletes and other employees of loud sports stadiums), there is no reason to further delay the adoption of noise limits in a much broader range of settings than just the factory floor and to neglect educating young people about the potential dangers of frequent exposure to loud music and leisure noise.

## Figures and Tables

**Table 1:** Organizations advocating for hearing conservation.

Name of Organization Advocating for Hearing Conservation	Web Address
Academy of Doctors of Audiology	audiologist.org *
Action on Hearing Loss	actiononhearingloss.org.uk *
American Academy of Audiology	audiology.org *
American Academy of Otolaryngology—Head and Neck Surgery	entnet.org *
American Speech-Language-Hearing Association	asha.org *
American Tinnitus Association	ata.org *
Center for Hearing and Communication	chchearing.org *
Dangerous Decibels	dangerousdecibels.org *
Hearing Education and Awareness for Rockers	hearnet.com *
Hearing Industries Association	hearing.org *
Hearing Loss Association of America	hearingloss.org *
Howard Leight	howardleight.com *
National Hearing Conservation Association	hearingconservation.org *
National Institute for Occupational Safety and Health	cdc.gov/niosh/topics/noise/ *
National Institute on Deafness and Other Communication Disorders	nidcd.nih.gov *
Sight & Hearing Association	sightandhearing.org *
The Hearing Conservation Workshop	heartomorrow.org *
The Noise Pollution Clearinghouse	nonoise.org *
The Quiet Coalition	thequietcoalition.org *

* Accessed on 15 April 2021.

## Data Availability

Not applicable.
